# Translation and evaluation of psychometric properties of the Amharic pediatric quality of life inventory 4.0 generic core scale for children with cancer

**DOI:** 10.1186/s12955-022-02077-8

**Published:** 2023-01-30

**Authors:** Tenaw Gualu Melesse, Janita Pak Chun Chau, William Ho Cheung Li, Kai Chow Choi, Mulugeta Ayalew Yimer, Abdulkadir Mohamed Said Gidey

**Affiliations:** 1 Nethersole School of Nursing, Faculty of Medicine, The Chinese University of Hong Kong, Hong Kong, Hong Kong SAR; 2 Department of Pediatrics and Child Health Nursing, College of Health Sciences, Debre Markos University, Debre Markos, Ethiopia; 3 Pediatric Hematology-Oncology Unit, Department of Pediatrics and Child Health, School of Medicine, University of Gondar, Gondar, Ethiopia; 4 Pediatric Hematology-Oncology Division, Department of Pediatrics and Child Health, College of Health Sciences, Addis Ababa University, Addis Ababa, Ethiopia

**Keywords:** Cancer, Child, Health-related quality of life, Quality of life, Psychometrics, Reliability, Validity

## Abstract

**Background:**

Childhood cancer negatively impacts a child's physical, mental, and behavioural health and significantly affects their health-related quality of life. The Pediatric Quality of Life Inventory 4.0 Generic Core Scale (PedsQL™ 4.0 GCS) is one of the most commonly used measures of the quality of life in children. However, the Amharic version of PedsQL™ 4.0 GCS has not been validated in a paediatric oncology population. This study aimed to translate and evaluate the psychometric properties of the Amharic PedsQL™ 4.0 GCS (PedsQL™ 4.0 GCS (A)) for Ethiopian children with cancer.

**Methods:**

A descriptive cross-sectional study was conducted among children aged 8–18 years with any type of cancer across the cancer trajectory. Cronbach’s alpha and intraclass correlation coefficient were computed to determine the internal consistency and test-retest reliability of the scale. The convergent validity was established by examining the correlation of the PedsQL™ 4.0 GCS (A) with the Amharic version of the Revised Child Anxiety and Depression Scale (RCADS-25(A)). Factorial validity was evaluated by conducting a confirmatory factor analysis.

**Results:**

The study included 142 participants with childhood cancer. PedsQL™ 4.0 GCS (A) had good validity and reliability. It demonstrated high internal consistency with a Cronbach’s alpha of 0.96 for the scale and 0.82–0.95 for the subscales. The intraclass correlation coefficient for the scale was 0.9 and that for the subscales was 0.76–0.90. The PedsQL™ 4.0 GCS (A) was highly correlated with RCADS-25 (A) (r = − 0.97, *p* < 0.001), supporting its convergent validity. The four-factor structure of the model fitted the data satisfactorily (χ^2^/df = 1.28; CFI = 0.97; TLI = 0.97; RMSEA = 0.05; SRMR = 0.05), supporting the factorial validity of the PedsQL™ 4.0 GCS (A).

**Conclusion:**

The PedsQL™ 4.0 GCS (A) demonstrates desirable psychometric properties for assessing quality of life among Ethiopian children with cancer. The scale can be used in clinical settings for assessing and evaluating quality of life in children with cancer. The use of parent-report versions and studies in those with different health conditions and healthy populations are necessary to further establish the psychometric properties of the PedsQL™ 4.0 GCS (A).

## Background

Childhood cancer is a leading cause of total cancer burden globally, causing significant mortality and morbidity. Globally, approximately 11.5 million disability-adjusted life years (DALYs) are estimated to have been caused by childhood cancer in 2017 [1]. The number of children diagnosed with cancer in Ethiopia each year exceeds 6000 [2].

Childhood cancer impedes a child’s physical, psychosocial and behavioural health [3], and significantly affects their quality of life (QOL) [4]. Children undergoing cancer treatment frequently report impaired QOL [5–7]. In a previous study, approximately 76%, 43%, and 39% of patients undergoing consolidation therapy reported physical, social, and emotional problems [8]. QOL impairment also adversely affects physical, psychosocial, emotional, and school functioning [9, 10] as well as causing increased pain [11, 12], fatigue [11, 12], symptoms of distress [13, 14], and functional impairment [15, 16].

Psychosocial assessment of children with cancer is strongly recommended as a standard of care in paediatric oncology [17]. Through systematic screening using appropriate instruments, specific problems can be identified and evidence-based interventions can be planned and implemented [18].

The Pediatric Quality of Life Inventory 4.0 Generic Core Scale (PedsQL™ 4.0 GCS) is one of the most commonly used measures of QOL in children [19]. It helps to evaluate QOL among children and adolescents with different health conditions [20]. It contains 23 items with four dimensions, including physical functioning (8 items), emotional functioning (5 items), social functioning (5 items), and school functioning (5 items). The PedsQL™ 4.0 GCS has well-established validity and reliability among children in the original study [20]. It has been translated into over 100 languages and shows acceptable psychometric properties in most of the studies in paediatric oncology [21–23], though other studies have raised concerns about some of its psychometric properties [24, 25].

To date, there is limited published evidence on the QOL of Ethiopian children with cancer. Thus, this study aimed to translate and evaluate the psychometric properties (including internal consistency, test-retest reliability, convergent validity, and factorial validity) of the Amharic child-report Amharic PedsQL™ 4.0 GCS among children with cancer in Ethiopia.

## Methods

### Study design

This study was part of a research project that examined the psychometric properties of different psychosocial measures in paediatric oncology. The study employed a descriptive cross-sectional methodology. It included two phases: (1) translation of the English version of PedsQL™ 4.0 GCS into Amharic and evaluation of the content validity of the translated version and (2) evaluation of the psychometric properties, including internal consistency, test-retest reliability, convergent validity, and factorial validity of the PedsQL™ 4.0 GCS (A).

#### Phase I: translation and content validation

We obtained permission to translate and validate the PedsQL™ 4.0 GCS from Mapi Research Trust on behalf of the original author. We chose the generic scale because there is an Amharic translation of PedsQL 4.0 GCS available from Mapi Research Trust, but no reports of reliability and validity were found in paediatric oncology. Moreover, Mapi was not involved in the linguistic validation process of this language version. Thus, we did the translation ourselves by adopting the model of back-translation, which includes forward-translation, review of the translated version, back-translation and review of translation equivalence [26], and compared the translated version with the original English version. Our first step was to invite a bilingual paediatric nurse who had experience with paediatric cancer patients in Ethiopia to translate the original English version of PedsQL™ 4.0 GCS into Amharic. An Amharic teacher then reviewed the translated version's wording and comprehension and refined some of the words to make them easier to understand. After that, another bilingual translator with a paediatric nursing background blindly back-translated the Amharic translation version to English. Whenever there was a discrepancy between the two versions (back-translated and original English versions), the principal investigator (PI) invited the bilingual reviewers to discuss and make revisions accordingly. Finally, the PI assessed the content and semantic equivalence by comparing the newly translated and existing Amharic versions [27]. The Likert scale rating and some items in the existing Amharic version (items 1, 3, 4, 7 and 8, 2 and 5, 3 and 5, and 3 and 5 in the physical, emotional, social and school functioning sub-scales respectively) were found to have translation inequivalence. Upon reviewing the two Amharic versions, our bilingual research team concluded that the new Amharic version (PedsQL™ 4.0 GCS (A)) achieved better semantic equivalence with the original English version. For example, in the existing Amharic version, the item “I have low energy” was translated as “I have very low energy”. The unnecessary added word “very” was removed from the item in the current translation. Similarly, in some parts of the existing Amharic version, the Likert scale “often” and “almost always” were translated as “almost never” and “sometimes” respectively which are different from the original English version. In the current study, we achieved translation equivalence for these and other items.

A panel of six bilingual experts, including a paediatric oncologist, an oncologist nurse, a psychiatric nurse, a nurse researcher, a psychologist and a paediatrician with clinical and research experience further evaluated the appropriateness of the translation and the content and cultural relevance of each item of the PedsQL™ 4.0 GCS (A) to QOL of children with cancer. The experts rated the content validity using a four-point Likert scale (1 = not relevant, 2 = somewhat relevant, 3 = quite relevant, and 4 = highly relevant) [28], and checked the comprehensiveness of the construct being measured. The content validity was assessed by computing the item level content validity index (I-CVI) and scale level content validity index (S-CVI). The I-CVI was determined by the proportion of experts who rated items 3 or 4, and the S-CVI was determined by the average of I-CVI for all items [29]. After that, 10 children with cancer with a mean age of 12.1 (3.1) were randomly recruited from one of the study hospitals and invited to assess the appropriateness of the items (understandability of the items by patients of their age group) and the time required to complete PedsQL™ 4.0 GCS (A).

#### *Phase II: evaluation of the psychometric properties of PedsQL™ 4.0 GCS (A)*

### Setting and participants

This study was conducted in four specialised hospitals in Ethiopia. The study participants were recruited from paediatric hematology/oncology outpatient and inpatient departments from January to April 2022. The inclusion criteria were (1) children aged eight to eighteen years old, (2) diagnosed with any type of cancer and at any stage of the cancer trajectory, (3) able to communicate in Amharic, (4) able to complete the PedsQL 4.0 GCS questionnaire themselves (grades 3 and above) and (5) able to provide oral child assent and written parent consent.

The sample size was determined based on a rule of thumb for sample size requirement of factor analysis. Using a minimum requirement of a 5:1 subject-to-item ratio [30], and considering a 10% non-response rate, a minimum of 128 participants were targeted to be recruited, excluding those who participated in evaluating the content validity. The participants were recruited using a consecutive sampling method until the required sample size was reached.

## Measurements

The data collection questionnaires comprise sociodemographic and clinical data sheets, PedsQL™ 4.0 GCS (A) and the Amharic version of the Revised Child Anxiety and Depression Scale (RCADS-25 (A)). Sociodemographic data including age, gender, educational status, religious affiliation, and residential address, and clinical data, including type of cancer diagnosis, time since diagnosis, types of therapeutic regimen received, and duration of treatment were collected.

PedsQL™ 4.0 GCS was used to measure HRQOL. The scale is available for different age groups (for 2–18-year-old children). In this study, we used a child report PedsQL™ ages (8–12 and 13–18). Each item was rated on a five-point Likert scale (0 = never, 1 = almost never, 2 = sometimes, 3 = often, and 4 = almost always). The total score and subscale scores were all linearly transformed to the range of 0–100. A higher score indicated better HRQOL [31]. The child report PedsQL™ 4.0 GCS had strong validity and high reliability (α = 0.88) in the original study [20]. The scale has also been translated and validated in different languages for children with cancer [21].

The convergent validity of PedsQL™ 4.0 GCS (A) was determined by correlation between PedsQL™ 4.0 GCS (A) and RCADS-25 (A). The RCADS-25 (A) was used to evaluate anxiety and depression. It contains 15 items in the anxiety subscale and 10 items in the depression subscale that were rated on a four-point Likert scale (0 = never, 1 = sometimes, 2 = often and 3 = always) [32]. The total score for each subscale and the total scale were computed by the sum of the score of items that comprise each subscale and the total scale respectively. To compute the total score, the total row scores were converted into T-scores, with T scores of ≥ 65–70 and ≥ 70 showing borderline clinical threshold and above clinical threshold respectively [33]. The RCADS-25 had good reliability and validity (α = 0.91 for the total anxiety scale and α = 0.80 for the depression scale) among clinical samples in the original version [34]. We have validated RCADS-25 (A) (α = 0.96) [unpublished data]. RCADS-25 is freely available for research use, and we obtained permission from the original author for translation and validation in our study.

### Data collection procedure

A research assistant (a nurse with a bachelor's degree and previous research experience) approached potential study participants and their parents during their regular medical appointments and at their inpatient beds to determine their eligibility. Assent was obtained from the child and informed written consent was obtained from the parent or legal guardian after the study was explained to them. In addition to obtaining clinical data from participants' medical records, participants were asked to complete the sociodemographic questionnaire, the PedsQL™ 4.0 GCS (A), and the RCADS-25 (A). To determine the test-retest reliability of the PedsQL™ 4.0 GCS (A), the same scale was administered among 50 randomly selected children two weeks after the initial data collection [35].

### Data analysis

Data were entered by using EpiData (version 3.1) and analysed using IBM SPSS 26 (IBM Corp. Armonk, NY, USA). The normality of variables with continuous data was assessed based on their skewness and kurtosis statistics. The demographic information and clinical data were summarised using appropriate descriptive statistics, including frequency, percentage, mean and standard deviations. The incomplete items were imputed on a scale by the mean of the completed items [31].

The content validity of PedsQL™ 4.0 GCS (A) was assessed using content validity index. The S-CVI score of 0.9 and I-CVI score of 0.78 are considered acceptable [29]. The internal consistency was assessed by calculating Cronbach’s alpha (α). An α of > 0.7 is considered acceptable [36]. The test-retest reliability was examined by computing the intraclass correlation coefficient (ICC). ICC was determined using a single measurement, absolute agreement, and 2-way mixed effects model, with a value of 0.75–0.9, and > 0.90 indicating good and excellent reliability respectively [37].

Convergent validity was evaluated by assessing the correlations between PedsQL™ 4.0 GCS (A) and RCADS (A) and their subscales. Evidence shows that QOL had a moderate to strong negative correlation with depression and anxiety [38, 39]. A correlation coefficient of 0.40–0.69, 0.70–0.89 and ≥ 0.9 are considered moderate, strong and very strong correlations respectively [40].

A confirmatory factor analysis was perform ed using IBM SPSS Analysis of a Moment Structures (AMOS) version 23 to examine the goodness-of-fit of the four-factor structure of PedsQL™ 4.0 GCS identified in the original study [20]. The parameters were estimated using the maximum likelihood estimation method. The multivariate outliers were checked using Mahalanobis distance at *p* < 0.001, and a multivariate normality was considered plausible when the absolute value of skewness and kurtosis lie between − 3 and + 3, and − 10 to + 10, respectively [41]. The goodness-of-fit of the model fit was evaluated by using various commonly used fit indices including χ2 statistic to the degree of freedom ratio (χ2/df ≤ 3), comparative fit index (CFI) ≥ 0.95, root mean square error of approximation (RMSEA) ≤ 0.08, Non-normed Fit Index (NNFI) also known as Tucker–Lewis index (TLI) ≥ 0.95 and standardized root mean square residual (SRMR) ≤ 0.1 [42].

## Results

### Content of PedsQL™ 4.0 GCS

The I-CVI ranged from 0.83 to 1 and its S-CVI was 0.91 indicating PedsQL™ 4.0 GCS (A) had good content validity. The expert panels recommended no revision or deletion of the items. Additionally, none of the participants who evaluated the content validity reported difficulty completing the PedsQL™ 4.0 GCS (A); thus, no revision of the items was required.

### Evaluation of the psychometric properties of PedsQL™ 4.0 GCS

#### Participant characteristics

A total of 142 participants who met the inclusion criteria consented to participate in this study. All the consented participants completed the questionnaires (response rate: 100%). The demographic and clinical characteristics of the participants are presented in Tables 1 and 2.

**Table 1:** Demographic characteristics of the participants (n = 142)

Participant characteristics		n (%)
Age (mean age = 11.6 (3.2))	8–12 years	85 (59.9)
	13–18 years	57 (40.1)
Gender	Male	82 (57.7)
	Female	60 (42.3)
Education	Primary school (Grades 3–8)	120 (84.5)
	Secondary school (Grades 9–12)	22 (15.5)
Religion	Christian	124 (87.3)
	Muslim	18 (12.7)
Residence	Rural	99 (69.7)
	Town	43 (30.3)
Family monthly income (Ethiopian birr/USD)	< 2500 (< 48.1)	48(33.8)
	≥ 2500–5000 (≥ 48.1–96.2)	61 (43.0)
	≥ 5000–10,000 (≥ 96.2–192.3)	26(18.3)
	≥ 10,000(> 192.3)	7(4.9)

1 USD ~ 52 Ethiopian birr

**Table 2:** Clinical characteristics of the participants (n = 142)

Clinical characteristics		n (%)
Cancer diagnosis	Hematological malignancies	94 (66.2)
	Wilms’tumor	13 (9.2)
	Rhabdomyosarcoma (RMS)	9 (6.3)
	Retinoblastoma	8 (5.6)
	Osteosarcoma	7 (4.9)
	Ewing Sarcoma	6 (4.2)
	Others*	5 (3.5)
Time since diagnosis	< 6 months	78(54.9)
	≥ 6–12 months	34 (23.9)
	≥ 12 months	30 (21.1)
Chemotherapy status	Not started	9 (6.3)
	On treatment	128 (90.1)
	Completed	5 (3.5)
Treatment regimens	Chemotherapy alone	110 (82.7)
	Chemotherapy + surgery	20 (15.0)
	Chemotherapy + radiation	2 (1.5)
	Chemotherapy + radiation + surgery	1 (0.8)
Duration on treatment	< 6 months	76 (57.1)
	≥ 6–12 months	30 (22.6)
	≥ 12–60 months	27 (20.3)

^*^Neuroblastoma, skin cancer and soft tissue sarcoma

#### Reliability

The Cronbach’s α of the PedsQL™ 4.0 GCS (A) was 0.96. The Cronbach’s α was 0.95, 0.82, 0.90 and 0.87 for the physical, emotional, social and school functioning subscales, respectively. The Cronbach’s α was above the minimum recommended level, indicating acceptable internal consistencies of the subscales and the scale.

The ICC of PedsQL™ 4.0 GCS (A) was 0.90 (95%CI, 0.79–0.95). The ICC for the subscales were 0.90 (95%CI, 0.83–0.94), 0.76 (95%CI, 0.61–0.86), 0.87 (95%CI, 0.78–0.92) and 0.86 (95%CI, 0.76–0.92) for the physical, emotional, social and school functioning subscales respectively. The results show good test-retest reliability of the subscales and the scale.

### Convergent validity

The convergent validity of the PedsQL™ 4.0 GCS (A) was evaluated by its correlation with RCADS-25 (A). PedsQL™ 4.0 GCS (A) and its subscales significantly correlated with RCADS- 25 (A) and its subscales. PedsQL™ 4.0 GCS (A) had a statistically significant negative correlation with RCADS-25 (A) (r = − 0.97, *p* < 0.001). It also had a significant negative correlation with anxiety subscales of RCADS-25 (A) (r = − 0.92, *p* < 0.001) and a negative correlation with depression subscales of RCADS-25 (A) (r = − 0.86, *p* < 0.001). Additionally, the correlation between subscales of PedsQL™ 4.0 GCS (A) and the anxiety subscale and depression subscale of RCADS-25 (A) ranged from (r = − 0.77 to − 0.92, *p* < 0.001) and (r = − 0.69 to − 0.79, *p* < 0.001) respectively.

### Confirmatory factor analysis

**Fig. 1:**
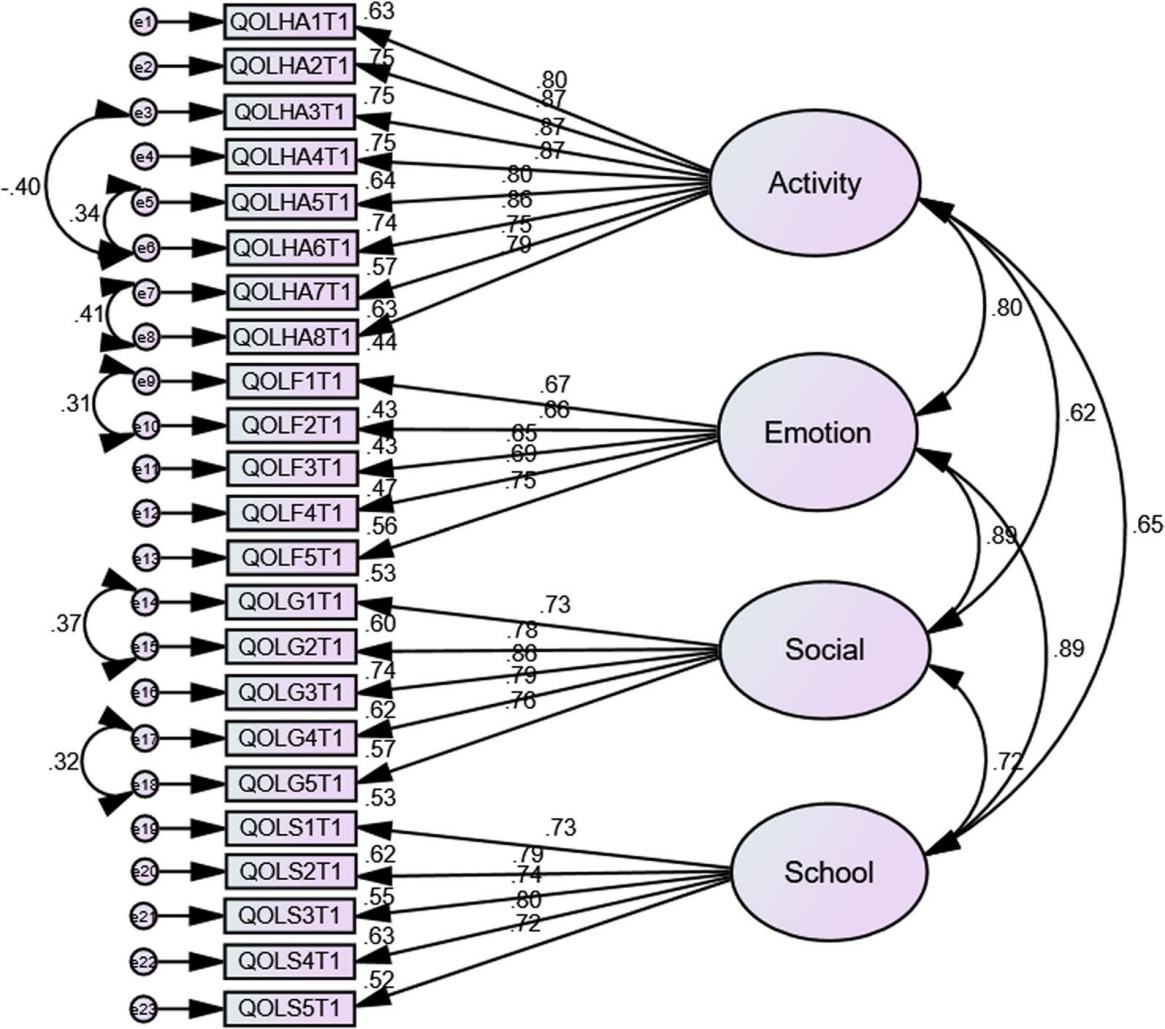
Standardized coefficient model of CFA

A confirmatory factor analysis (CFA) was conducted to determine the five-factor structure of PedsQL™ 4.0 GCS (A) identified in the original study [20]. The multivariate outliers were screened through Mahalanobis distance at *p* < 0.001[41] and all the observations have a *p*-value of > 0.007, indicating no significant multivariate outliers detected. Although the individual absolute Kurtosis and skewness values are within the normal range [41], the joint multivariate kurtosis value was found to be 30.98 with a critical ratio of 5.44. Thus, to address this violation of multivariate normality, the Bollen-Stine bootstrap test with 10,000 bootstrapped samples was used to evaluate the overall goodness-of-fit of the null model. To further improve the fit of the model, the covariance paths were added within the same construct when a modification index (MI) was matched with a large, expected parameter change. The factor loadings of each item ranged from 0.65 to 0.87 (Fig. 1) and were in the acceptable ranges for the sample size included in this study [43]. The bootstrapped test showed a non-significant result for the null model (*p* = 0.229), indicating improved model fit. The four-factor model of PedsQL™ 4.0 GCS (A) fit the data as evaluated by different indices (χ^2^/df = 1.28; CFI = 0.97; TLI = 0.97; RMSEA = 0.05; SRMR = 0.05).

## Discussion

The purpose of this study was to translate and evaluate the psychometric properties (including internal consistency, test-retest reliability, convergent validity, and factorial validity) of Amharic PedsQL™ 4.0 GCS (A) for children with cancer.

The results generated from this study support the reliability and validity of PedsQL™ 4.0 GCS (A) for Ethiopian children with cancer. The scale also demonstrated good content validity. The results show high internal consistency, with Cronbach’s α of 0.96 for the overall scale and 0.82–0.95 for the subscales. The results are higher than the minimum acceptable Cronbach’s α of 0.7 [36] and consistent with the original study [20], indicating high internal consistency of the scale.

The results also revealed good test-retest reliability of the scale and subscales. The ICC of the scale was 0.90 and the ICC for the subscales ranged from 0.76 to 0.90 which was higher than the minimum acceptable value of 0.75 [37], and congruent with previous studies that reported acceptable test-retest correlations [23, 44, 45]. Higher scale test-retest reliability indicates higher test stability over time [46, 47].

Our study also demonstrated that PedsQL™ 4.0 GCS (A) and its subscales were strongly correlated with RCADS-25 (A) and its subscales. The results are in line with previous studies which demonstrated that QOL has a moderate to strong correlation with distress symptoms [38, 39]. The strong correlations indicate high convergent validity, confirming that the constructs expected to be theoretically related are indeed related [48, 49].

The confirmatory factor analysis showed that the model satisfactorily fit the data and provided strong evidence of the scale’s factorial validity. The result supported the four-factor structure reported in the original study [20].

However, it’s important to acknowledge the limitations of this study. This study is part of a large study that validated different measures for children in paediatric haematology-oncology. Thus, the criterion-related validity of PedsQL™ 4.0 GCS (A) was evaluated by comparing it with the other measure i.e., RCADS-25(A), which is not actually measuring the same construct but is theoretically related. The use of two measures without previously established validity and reliability to validate each other may affect the criterion validity of the scale. Additionally, we did not evaluate the parent report PedsQL™ 4.0 GCS and compared it with the child’s self-report PedsQL™ 4.0 GCS (A) to identify a more appropriate measure.

### Clinical and research implications

This validation of PedsQL™ 4.0 GCS in Amharic, the most widely spoken and written language in Ethiopia [50], is of paramount importance. The strong psychometric properties of the PedsQL™ 4.0 GCS (A) indicate its cultural relevance. Thus, the scale could be used to assess the QOL of children with cancer in clinical settings and for research purposes.

As validating the scale in clinical samples of paediatric cancer patients cannot be generalised to other health conditions and non-clinical samples, validating the scale in different health conditions and the community settings such as schools is warranted. Further validation of the scale in other commonly used languages in Ethiopia would be helpful.

## Conclusion

The PedsQL™ 4.0 GCS (A) demonstrates good reliability and validity in evaluating the QOL for Ethiopian children with cancer. The scale can be used in clinical settings for assessing and evaluating quality of life in children with cancer. Including parent report versions and studies in different health conditions and healthy populations, such as schools, are warranted to further establish the psychometric properties of the PedsQL™ 4.0 GCS (A).

## Data Availability

The data set used during the current study can be obtained from the corresponding author on reasonable request.
